# Tirofiban, a Glycoprotein IIb/IIIa Antagonist, Has a Protective Effect on Decompression Sickness in Rats: Is the Crosstalk Between Platelet and Leukocytes Essential?

**DOI:** 10.3389/fphys.2018.00906

**Published:** 2018-07-11

**Authors:** Kate Lambrechts, Sébastien de Maistre, Jacques H. Abraini, Jean-Eric Blatteau, Jean-Jacques Risso, Nicolas Vallée

**Affiliations:** ^1^Département Environnement Opérationnel, Unité Environnements Extrêmes, Institut de Recherche Biomédicale des Armées – Equipe Résidente de Recherche Subaquatique Opérationnelle (Armed Forces Biomedical Research Institute – Resident Operational Subaquatic Research Team), Toulon, France; ^2^Laboratoire Motricité Humaine Expertise Sport Santé (LAMHESS – Human Motricity, Education, Sport and Health Laboratory), Université du Sud Toulon Var, La Garde, France; ^3^Hôpital d’Instruction des Armées – Service de Médecine Hyperbare et Expertise Plongée (Military Teaching Hospital – Hyperbaric Medicine and Diving Expertise Department), Toulon, France; ^4^Département d’Anesthésiologie, Université Laval, Laval, QC, Canada; ^5^Faculté de Médecine, Université de Caen Normandie (UNICAEN), Caen, France

**Keywords:** dive, ischemia, systemic inflammation, pressure, stroke, inert gas

## Abstract

In its severest forms, decompression sickness (DCS) may extend systemically and/or induce severe neurological deficits, including paralysis or even death. It seems that the sterile and ischemic inflammatory phenomena are consecutive to the reaction of the bubbles with the organism and that the blood platelet activation plays a determinant role in the development of DCS. According to the hypotheses commonly put forward, the bubbles could either activate the platelets by direct contact or be the cause of abrasion of the vascular epithelium, which would expose the basal plate glycogen and then prompt the platelets to activate. The purpose of this study is to confirm anti-platelet drugs specific to GPIIb/IIIa integrin could prevent DCS, using a rat model. There is a significant difference concerning the incidence of the drug on the clinical status of the rats (*p* = 0.016), with a better clinical outcome for rats treated with tirofiban (TIR) compared with the control rats (*p* = 0.027), even if the three anti-GPIIb/IIIa agents used have limited respiratory distress. TIR limited the decrease in platelet counts following the hyperbaric exposure. TIR help to prevent from DCS. TIR is specific to GPIIb/IIIa whereas eptifibatide and abciximab could inhibit αVβ3 and αMβ2 involved in communication with the immune system. While inhibiting GPIIb/IIIa could highlight a platelet-dependent inflammatory pathway that improves DCS outcomes, we wonder whether inhibiting the αVβ3 and αMβ2 communications is not a wrong approach for limiting mortality in DCS.

## Introduction

Systemic inflammatory response is a potentially serious problem because the triggering of inflammatory and pro-thrombotic inflammatory cascades, as well as endothelial damage, may cause multiple organic lesions (Smail et al., 1995). We have recently described a similar picture in our decompression sickness (DCS) animal model (Cosnard et al., 2017).

The presence of bubbles circulating at vascular level remains the *primum movens* at the origin of the decompression accident which regularly degenerates into DCS. In its severest forms, DCS may extend systemically and/or induce severe neurological deficits, including paralysis or even death. It seems that the ischemic and inflammatory phenomena are consecutive to the presence of intravascular bubbles, and that blood platelet activation plays a determinant role in the development of DCS. According to the hypotheses commonly put forward (Brubakk et al., 2003), the bubbles could either activate the platelets by direct contact or be the cause of abrasion of the vascular epithelium, which would expose the glycogen of the basal plate and then prompt the platelets to activate. Beyond the well-known thrombotic role of platelets, it would also be interesting to consider the involvement of the platelets in the immune response (Harifi and Sibilia, 2016). In fact, according to the work of Sibilia (2007), it would seem that DCS can be qualified as a disorder that is auto-inflammatory (it continues despite the disappearance of the bubbles), sterile (the air in the bubbles is not strictly speaking a pathogenic element) and it is neutrophilic in type (Chen and Nunez, 2010; Thom et al., 2015).

Previous work confirmed that the degree of platelets aggregation in the rat is a reliable index for the severity of DCS (Pontier et al., 2008). Our results also showed that clopidogrel (Plavix ©), a specific inhibitor of ADP receptors, limits occurrence, and severity of DCS by reducing the amount of platelet aggregation (Pontier et al., 2011). It was also noted that fluoxetine, which could have an anti-platelet aggregation role by blocking the platelet SERT receptors (Halperin and Reber, 2007), reduces the incidence of a provocative decompression (Blatteau et al., 2012, 2015; Vallee et al., 2016; Cosnard et al., 2017). More recently, other results have confirmed the involvement of platelet aggregation in the pathogenesis of DCS, by this time spotlighting a preventive and beneficial effect of abciximab (ABX; Reopro^®^), an anti-platelet drug of the anti-GPIIb/IIIa type (Lambrechts et al., 2015). These results suggest that the anti-GPIIb/IIIa agents may have a strong preventive effect.

The purpose of this study was to investigate the effects of the different anti-platelet drugs specific to GPIIb/IIIa integrin, approved by the Food and Drug Agency, ABX (Reopro^®^), tirofiban (TIR; Agrastat^®^), and eptifibatide (EPT; Integrilin^®^), and to assess the effects of them on the occurrence of DCS. Although these three substances have an action on activated platelet aggregation by blocking the fibrinogen inter-platelet bridge between the GPIIb/IIIa (Ma et al., 2007), these molecules will also have collateral actions. TIR and EPT imitate the amino acid sequence of the (Arg-Gly-Asp) RGB bond of the fibrinogen ligand, whereas ABX is an anti-β3 monoclonal antibody. TIR would be specific to GPIIb/IIIa, ABX, and EPT would have a wider mode of action by also linking on the αVβ3 and αMβ2 (CD11b/CD18, Mac-1) receptors present on the leukocytes (Simon et al., 1997; Bledzka et al., 2013). This would make conquering the inflammatory symptoms more effective (Coller, 1999a). Effectively, it should also be noted that the leukocyte count is also a reliable DCS index (Blatteau et al., 2012, 2015; Vallee et al., 2016; Cosnard et al., 2017). We hypothesize that these drugs should prevent from DCS by limiting the crosstalk between platelets and leukocytes and therefore limit their decreases observed during a pathogenic decompression.

## Materials and Methods

**FIGURE 1:**
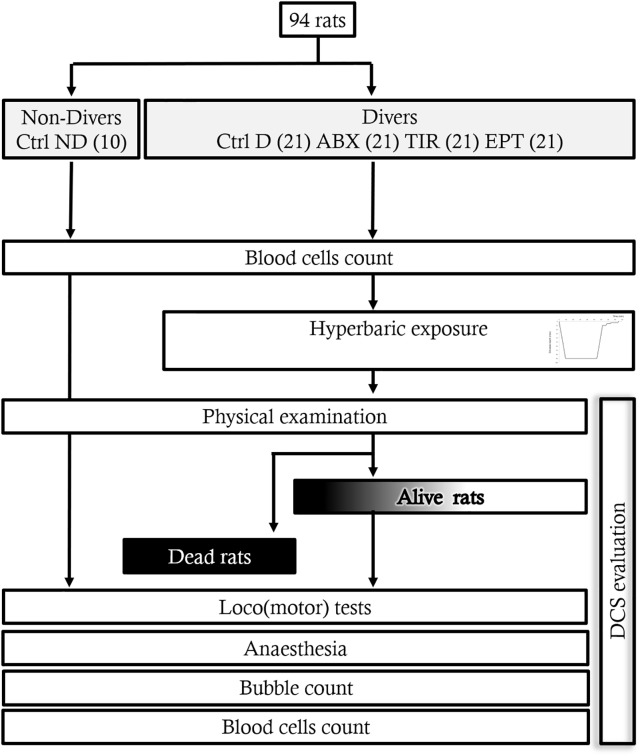
Flow chart of the experiment.

The experimental design can be followed on **Figure 1**.

### Animals and Ethics

All the procedures involving experimental animals comply with European Union rules (Directive 2010/63/EU) and French law (Decree 2013/118). The Institut de Recherche Biomédicale des Armées Ethics Committee approved these studies in 2016. The rats (Laboratoires Harlan, France), all adult males, are housed in an accredited animal housing facility at 22 ± 1°C with a regulated day/night cycle (day at 0600/night at 1800). They have access to drinks and food (kibble from Harlan Laboratories, 18% protein) *ad libitum*.

In agreement with our Ethics Committee, we took inspiration from the Swiss veterinary guide to establish a form for monitoring the welfare of the animals (Cosnard et al., 2017). A dedicated observer is responsible for scoring (from 0 to 3) the stress and pain felt by each animal: 0 corresponds to a 0° of stress and three is the maximum. A degree of stress of 3 in one case or a total score of 12 represents a criterion for stopping. The items refer to vocalization, licking, the presence of tears, aggression or withdrawn behavior, labored breathing, motor or locomotor disorders with paralysis for example. In this study, no score reached 12, and it was not necessary to have recourse to anticipated euthanasia. However, the animals presenting items with a score of 3 for convulsions die very quickly.

At the end of the experimentation, the animals were anesthetized by induction with isoflurane (Bellamont, first at 5% and then at 2%), in order to save time and minimize stress, then by intraperitoneal injection (1-ml syringe, Omnican^®^, B. Braun, Melsungen, Germany) of a mixture of ketamine (Imalgene^®^ 1000, 100 mg/kg, AstraZeneca, London, United Kingdom), acepromazine (Calmivet^®^, 1.65 mg/kg, Vétoquinol S.A., Lure, France) and xylazine (Rompun^®^ 2%, 16 mg/kg, Bayer HealthCare, KVP, Kiel, Germany).

### Drugs and Dosages

A total of 94 animals weighing 394.8 ± 14.9 g were used. The rats were separated into 5 groups: 10 control rats not exposed to the hyperbaric protocol and receiving a placebo (Ctrl ND), and 84 rats exposed to the hyperbaric protocol, 21 of which received the placebo (Ctrl D), 21 treated with ABX, 21 treated with TIR, and 21 others treated with EPT. The start of the hyperbaric protocol before 30 min, the anti-platelet drugs were injected (i.v. q.s 0.3 ml NaCl 0.9%) under gaseous general anesthesia (Isoflurane 5%, Bellamont, for 2 min) in the dorsal veins of the penis in the following concentrations: ABX (Reopro^®^ 2 mg/ml, Centocor B.V) at 1 mg/kg; TIR (Agrastat^®^ 250 μg/ml, Correvio Pharma Corp) at 60 μg/kg and, EPT (Integrilin^®^ 0.75 mg/ml, Glaxo Group Ltd.) at 180 μg/kg. For the two groups of the control rats (Ctrl ND and Ctrl D), a solution of NaCl is injected in the same way as the anti-platelet drugs.

### Hyperbaric Exposure

Batches of 8 freely moving rats (4 per cage and 2 per group), from Groups Ctrl D, ABX, TIR, and EPT, were subjected to the hyperbaric protocol which generates decompressions sickness in a 200 L caisson with three observation portholes.

The protocol has two compression speeds. The animals were subjected to an air compression procedure at a speed of 10 kPa.min^-1^ up to an absolute pressure of 200 kPa (corresponding to a depth of 10 m of seawater) and then a speed of 100 kPa.min^-1^ up to a pressure of 1000 kPa (corresponding to a depth of 90 msw) where they remained for 45 min. The rats were then decompressed at a speed of 100 kPa.min^-1^ up to 200 kPa, then a speed of 10 kPa.min^-1^ until return to normal pressure, adhering to 5 min stages at 200 kPa (10 msw) and 160 kPa (6 msw) and a final stage of 10 min at 130 kPa (3 msw). The decompression speed was automatically controlled by a computer connected to an Analog/Digital converter (NIUSB-6211, National Instrument, United States), itself connected to a solenoid valve (Belimo LR24A-SR, Switzerland) and a pressure transmitter (Pressure Transmitter 8314, Bürkert Fluid Control Systems, Germany). The program used to control the compression and decompression speeds was devised by a laboratory engineer at DASYLab (DASYLab National Instruments, United States). The compressed air was supplied by a diving compressor (Mini-Verticus III, Bauer Comp Holding, Germany) coupled to a 100-liter unit at 30 MPa, connected to a pressure relief valve (LTHS 400 0086, Alphagaz^TM^, Rousset, France). The oxygen analysis was performed using a micro fuel electrochemical cell (G18007 Teledyne Electronic Technologies/Analytical Instruments, United States). The CO_2_ produced by the animals was captured with soda-lime (< 300 ppm, GE Healthcare, Helsinki, Finland). The gases were mixed by a fan, and the temperature inside the caisson was measured with a heat probe (Pt 100, Eurotherm, France).

### Diagnoses and Behavioral Tests

The physical examination for the rats is established over a 30-min observation period with a collection of clinical signs, where the respiratory difficulties, motor disorders, convulsions, and death are referenced with a time index. Then, these observations are completed by more specific tests for (loco)motor disorders: the *beam-walk test from 1 to 7* (agility test on a 1.5-long and wide plank calibrated varying from 7.7 to 1.7 cm, and placed 1.1 m above the void) is practiced 2 weeks before the dive and after the dive. It involves allowing the rat to move on an ever-narrower board above the void. The *rollover test* consists of a simulated fall situation causing a reflex rollover in the animal so that it falls on its paws. It is assessed on a score of 0 to 2. The *toe-spreading reflex test* (Pockett and Gavin, 1985) assesses motricity and more especially the functional impairment of the sciatic nerve (SFI Index). It is based visually on the spreading of the toes where 2 is a normal state, 1 a weak spread, and 0 complete inability to spread the toes. This test is seconded by the diagnosis of motor impairment of the hind paws (MIHP), where a score of 5 indicates normal motricity, 4 a rat which limps, 3 a paw which is stretched and does not go spontaneously back into place, 2 a paw spontaneously to the rear, 1 a paw that no longer moves still capable of muscle contraction, and 0 an inert paw.

### Clinical Status

The Lethal DCS status encompasses rats that die in the hour following the end of the hyperbaric protocol. The DCS status was attributed when the rat presented serious neurological signs in the form of paresis or paralysis of at least one limb, convulsions and/or reduced performance in SFI, MIHP locomotor tests, with a *beam walk test* score reduced by at least 2 points. The other rats are considered to be No DCS.

### Anesthesia and Sacrifice

Coming out of the hyperbaric chamber after 60 min, all the animals were anesthetized by induction with isoflurane (Bellamont, first at 5% and then at 2%), then by intraperitoneal injection (1-ml syringe, Omnican^®^, B. Braun, Melsungen, Germany) of a mixture of ketamine (Imalgene 1000, 100 mg/kg, AstraZeneca, London, United Kingdom), acepromazine (Calmivet^®^, 1.65 mg/kg, Vétoquinol S.A., Lure, France) and xylazine (Rompun^®^ 2%, 16 mg/kg, Bayer HealthCare, KVP, Kiel, Germany).

At the end of the experiment, rats were sacrificed by an injection of sodium pentobarbital (200 mg/Kg IP; Sanofi, Paris, France).

### Detection of Circulating Bubbles

The Doppler detection of circulating bubbles in the heart is performed 60 min after the dive under general anesthetic with an echocardiograph (MicroMaxx Ultrasound System, Sonosite, Bothell, WA, United States) fitted with a 4–8 MHz probe, in 2D mode for the location of the right cavity of the heart and then in pulsed Doppler mode to detect the bubbles. The quantification of bubbles is performed using the Spencer score (Spencer, 1976), with a score of 0 in the absence of bubbles, 1 with several bubbles, 2 with bubbles at each cardiac revolution, and a score of 4 when the noise of the bubbles is continuous (score of 3) and covers the heart sounds.

### Blood Analyses

The blood counts are performed from 15-μl blood taken from the tip of the tail and diluted in the same volume of EDTA at 2 mM (Sigma, France). The analysis is performed using an automaton (Scil Vet abc, SCIL Animal care company, France) on samples taken 30 min before or 60 min after exposure to the hyperbaric protocol. The values for the second blood sample are corrected depending on the variation in the hematocrit.

### Cytokine Detection

Under anesthesia, blood samples were collected by an intra-aortic puncture to determine the values of plasmatic cytokine levels. Blood was collected into sterile 4-ml tubes containing lithium heparin (BD Vacutainer^®^, BD-Plymouth, United Kingdom) and, within 30 min, plasma was separated out by simple centrifugation at 1200 ×*g* and 4°C for 15 min. The supernatant was kept at –80°C until testing.

Inflammatory cytokines were assayed using a Bioplex100 (Bio-Rad Inc., CA, United States) and a series of rat ELISA kits: PF4, CD62P (P-selectin), IL-1α, IL-10, CD62L (L-selectin), ICAM-1, VCAM-1, integrin-β2, MIP-1α (CCL3), MCP-1 (CCL2), and BLC-1 (CXCL13) (ELISA Kit, Antibodies-online GmbH, Germany). Samples, standards, and quality controls were all run in duplicate. All standards and quality controls were made up as recommended by the supplier.

### Statistical Analyses

The blood count analyses are calculated according to an individual variation percentage. For the groups, the data is expressed in means and standard deviations. The difference is analyzed using Mann–Whitney *U*-test (MW) or Wilcoxon (W) test for paired comparisons. Multiple comparisons are performed using the Kruskal–Wallis test followed by a *post hoc* Dunn’s test. Accepted risk α is 5%.

## Results

### Effect of the Hyperbaric Exposure and Clinical Status of the Rats

Following exposure to the hyperbaric protocol, from 1.1 ± 2.6 min, the animals could present neurological impairments with convulsions, paralysis, paresis, sciatic functional impairment, or limping, as objectified by the tests. Respiratory distress was also observed within particular dyspnea, but no bleeding was observed in the sputum or nasal serosa, therefore, excluding pulmonary barotrauma.

In the most serious cases, the animals could die (Lethal DCS), after 3.5 ± 6.9 min on average. As a matter of fact, no biological analyses or bubble detection could have been performed on these animals.

**FIGURE 2:**
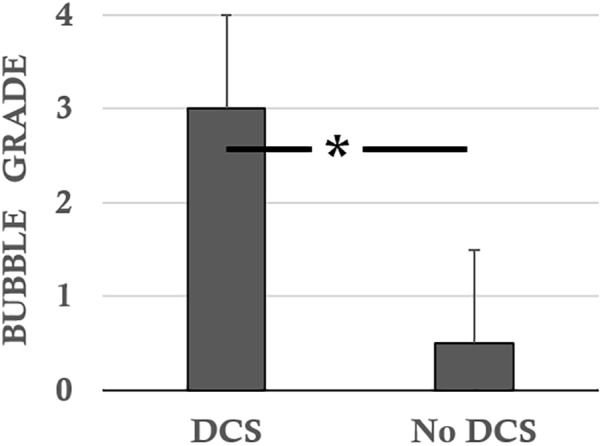
Bubble scores according to the status of the rats following the hyperbaric protocol. ^∗^ notes significant difference (*p* < 0.05).

The DCS rats (*n* = 7) presented scores of 2.0 ± 1.6 for the *Beam test*, 1.14 ± 1.07 for the rollover test, 0.57 ± 0.53 for functional impairment of the sciatic nerve and 2.43 ± 1.72 and 2.57 ± 1.81 for global motor impairment of the right and left hind paws (MIHP), respectively. Their bubble scores were 3.0 ± 0.0 (**Figure 2**).

The No DCS diver rats (*n* = 7) presented scores of 6.2 ± 0.6 for the *Beam test*, 1.79 ± 0.30 for the rollover test, 1.84 ± 0.15 for functional impairment of the sciatic nerve and 4.63 ± 0.15 for global MIHP indifferently. The bubble levels were 0.5 ± 1.1 (**Figure 2**).

These parameters are all significantly different when the different statuses (DCS, NoDCS) are compared with each other (i.e., beam and rollover tests, SFI, MIHP, bubbles grade; KW, *p* < 0.0001, Dunn_NoDCS/DCS_ *p* < 0.0001), all drug combined.

### Clinical Status According to Treatments

There is a significant difference, induced by the treatments, on the clinical status of the rats (KW_Abx/Tir/Ept/CtrlD/CtrlND_, *n* = 21/21/21/21/10, *p* = 0.016), in favor of a better clinical prognosis for rats treated with TIR compared with Ctrl D (*post hoc* Dunn’s test_TIR/CtrlD_ *p* = 0.027).

**FIGURE 3:**
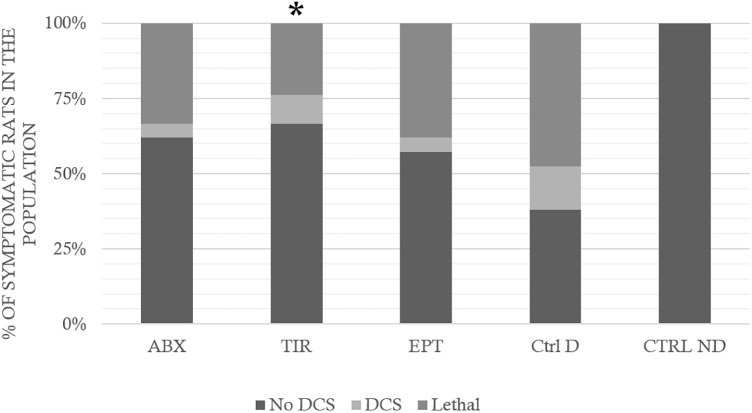
Clinical status after the hyperbaric protocol exposure. ABX: Abciximab; TIR: Tirofiban; EPT: Eptifibatide; Ctrl D: Control Dive; Ctrl ND: Control No Dive. The Lethal DCS status encompasses rats that die in the hour following the end of the hyperbaric protocol. The DCS status was attributed when the rat presented serious neurological signs in the form of paresis or paralysis of at least one limb, convulsions and/or reduced performance in SFI, AMPA locomotor tests, with a *beam walk test* score reduced by at least 2 points. The other rats are considered to be No DCS. ^∗^ notes significant difference (*p* < 0.05) in the occurrence of symptoms compared to the Ctrl D group.

In control rats exposed to the hyperbaric protocol (Ctrl D) (**Figure 3**), there was 48% mortality in 1 h (Lethal DCS), 17% of rats presenting non-lethal DCS symptoms (DCS) and 38% of rats were unscathed (No DCS).

In rats treated with ABX, 33% of animals died, 24% were classed as DCS, and 62% No DCS.

Among the rats treated with TIR, 24% of animals died, 10% were classed as DCS, and 67% No DCS, and in those treated with EPT, 38% of animals died, 5% were classed as DCS, and 38% No DCS.

More precisely, treated rats (ABX, TIR, or EPT) displayed significantly less respiratory distress than the control rats (Ctrl D) (KW_Abx/Tir/Ept/CtrlD/CtrlND_, *n* = 21/21/21/21/10, *p* = 0.030; Dunn_ABX/CtrlD_ *p* = 0.006; Dunn_TIR/CtrlD_ *p* = 0.006; Dunn_EPT/CtrlD_ *p* = 0.036). The treatment has no significant impact on the other clinical signs. There is no significant difference, which could be attributed to the effect of a drug, in the time of appearance of the first symptoms (from 1.1 ± 2.6 min) or death (3.5 ± 6.9 min), that is to say, treatments did not just delay the onset of symptoms, they limited their occurrence. There was no significant difference on the bubble grade that could be linked to the treatment.

### Blood Analyses

#### Blood Cell Counts

**FIGURE 4:**
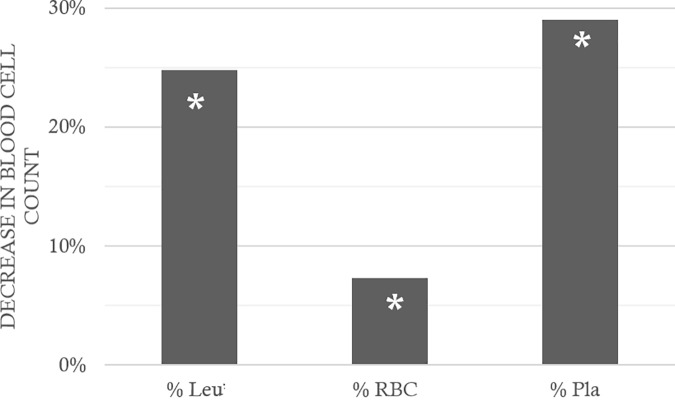
Fall in blood cells count, between before and after the hyperbaric exposure. Leu: leukocytes; RBC: Red Blood Cells; Pla: Platelets. As a result showed hemoconcentration linked to the hyperbaric exposure, the blood parameters registered after the dive was corrected individually depending on the variation in the hematocrit, and then compared to the first blood punctures to present their changes (%) on the histogram. ^∗^ notes significant difference (*p* < 0.05) between before and after the dive for the same parameter.

**FIGURE 5:**
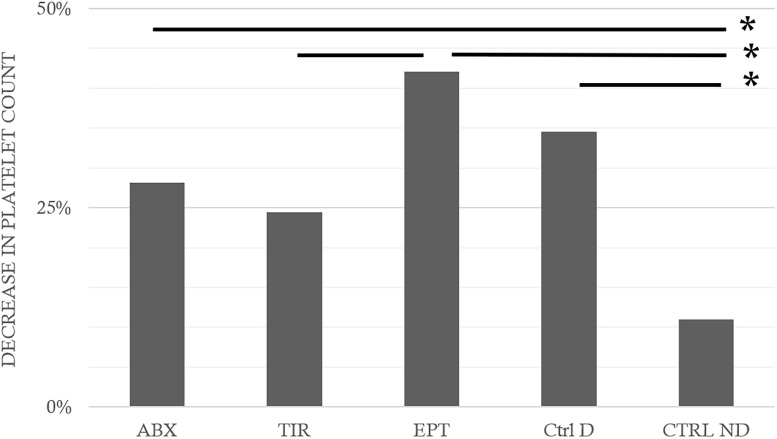
Fall in platelet counts following the hyperbaric exposure according to the treatment groups. ABX: Abciximab; TIR: Tirofiban; EPT: Eptifibatide; Ctrl D: Control Dive; Ctrl ND: Control No Dive. ^∗^ notes significant difference (*p* < 0.05) between groups.

Following the hazardous protocol, the blood parameters varied in all diving animals, without distinction. Thus, in surviving animals that could be punctured, the hematocrit had increased on average by 24.2 ± 70.8% (Wilcoxon, *n* = 62, *p* = 0.010). As this result showed hemoconcentration linked to the hyperbaric exposure, the blood count parameters were corrected individually depending on the variation in the hematocrit. The non-diving animals presented significant differences compared with the divers, particularly with the Ctrl D and EPT groups (KW_Abx/Tir/Ept/CtrlD/CtrlND_, *n* = 21/21/21/21/10; *p* = 0.043, Dunn_CtrlND/CtrlD_ *p* = 0.020; Dunn_CtrlND/EPT_ *p* = 0.018). EPT does not seem to have made it possible to limit hemoconcentration even if no inter-group difference appeared between the divers. Similarly (**Figure 4**), the counts of leukocytes, red blood cells, and platelets decreased (after correction) by 24.8 ± 64.5, 7.3 ± 15.5, and 29.0 ± 31.5%, respectively. This was significant after the dive (Wilcoxon, *n* = 62, *p* < 0.0001; *p* < 0.0001; *p* < 0.0001), with a significant inter-group difference for the platelets alone (KW_Abx/Tir/Ept/CtrlD/CtrlND_, *n* = 21/21/21/21/10; *p* = 0.017, *Post hoc* testes: Dunn_CtrlND/CtrlD_ *p* = 0.023; Dunn_CtrlND/ABX_ *p* = 0.019; Dunn_CtrlND/EPT_ *p* = 0.001; Dunn_EPT/TIR_ *p* = 0.016). Within these reductions (**Figure 5**), significant differences are noted in the proportions, between the Ctrl ND (11.0 ± 24.1%) and EPT (42.1 ± 25.1%) or ABX (28.2 ± 44.6%), and between EPT and TIR; among the divers, TIR was the group where the number of circulating platelets had reduced least 24.4 ± 26.1% (Ctrl D: 34.6 ± 25.1%). It should, therefore, be remarked that TIR seemed to limit the reduction in the number of circulating platelets, i.e., the platelet recruitment.

Hematocrit is calculated according to the Red Cell Count and the Mean Corpuscular Volume (MCV), both being measured directly by the automate. This result is the consequence of the decrease in Red Cell Count on one hand (RBC; Wilcoxon, *n* = 62, *p* < 0.0001, or W_Abx/Tir/Ept/CtrlD_, *n* = 17/17/15/13; *p*_Abx_= 0.016; *p*_Tir_= 0.037; *p*_Ept_= 0.003; *p*_CtrlD_= 0.012), and the increase of the MCV on the other hand, whatever is the drug considered (MCV; Wilcoxon, *n* = 62, *p* = 0.010, or W_Abx/Tir/Ept/CtrlD_, *n* = 17/17/15/13; *p*_Abx_= 0.016; *p*_Tir_= 0.037; *p*_Ept_= 0.003; *p*_CtrlD_= 0.012). These MCV variations, between before and after the dive, are not encountered in non-diving animals (W_CtrlND_: *n* = 10, *p* = 0.953). Actually, there is no significant difference between group, concerning the MCV after the dive (KW: *p* = 0.090), nor before (KW: *p* = 0.590).

#### Circulating Cytokines and Chemokines

BLC-1 levels tended to be significantly different between treatment groups (KW_Abx/Tir/Ept/CtrlD/CtrlND_, *n* = 21/21/21/21/10; *p* = 0.080), with significant higher levels revealed in *post hoc* testes in the TIR Group compared to the Ctrl ND and to the Ctrl D group to a lesser extent (*Post hoc* testes for BLC-1: Dunn_CtrlND/TIR_ *p* = 0.009; Dunn_CtrlD/TIR_ *p* = 0.049; Dunn_CtrlND/EPT_ *p* = 0.035). No significant difference emerged with respect to others cytokines analyzed in arterial blood [KW_Abx/Tir/Ept/CtrlD/CtrlND_; PF4 *p* = 0.757; CD62P (P-selectin) *p* = 0.418; IL-1α *p* = 0.519; IL-10 *p* = 0.936; CD62L (L-selectin) *p* = 0.421; ICAM-1 *p* = 0.118; VCAM-1 *p* = 0.464; integrin-β2 *p* = 0.386; MIP-1α (CCL3) *p* = 0.387; MCP-1 (CCL2) *p* = 0.423].

## Discussion

Like the previous studies, the dive protocol definitely produced DCS (Pontier et al., 2008; Blatteau et al., 2013, 2015; Vallee et al., 2013; de Maistre et al., 2016a; Cosnard et al., 2017), seen overall by an alteration in clinical and behavioral performances, particularly indicating neurological impairment, and accompanied by a reduction in the number of circulating leukocytes, red blood cells or platelets, and hemoconcentration. More specifically, the MCV increased in diving animals. It could suggest that older red blood cells disappeared or new reticulocytes were released in circulation from the spleen. It should be recalled that reticulocytes are slightly larger than mature erythrocytes, so that, overall, a raised MCV may be due to a larger number of these immature red cells. But the two are not opposed, especially in DCS where the most common theory refers to the destruction of cells by bubbles. As a concomitant result, the increase in the level of bubbles detected is linked to aggravation of the animal’s symptoms.

Among the 3 anti-GPIIb/IIIa molecules, only TIR changes the clinical status favorably, namely the clinical examination objectivized by the rats’ performance levels during the physical and behavioral tests, following exposure to the hazardous protocol. TIR prevents platelet recruitment, and is linked to an increase in BLC-1 concentration at the circulating level, compared to the other groups. If the anti-aggregation effect generally described for ABX and EPT (Bledzka et al., 2013) is considered, it leads us to suggest that the hyperbaric protocol alters their mode of action. This result confirms once again (Pontier et al., 2008, 2009, 2011; Lambrechts et al., 2014, 2015) that platelets play a major role in the occurrence of DCS, because stabilizing them by preventive drugs shows a significant effect on the appearance of DCS. However, the inability of these drugs to prevent the occurrence of these accidents completely indicated that other phenomena are involved in the genesis of DCS.

Activated platelets express adhesive proteins on their surfaces, including GPIIb/IIIa, to bind to the leukocytes. The resulting platelet/leukocyte complexes invite the platelets to release various alpha-granule proteins, encompassing coagulation factors, chemokines, and mitogenic factors (Diacovo et al., 1996; Evangelista et al., 1996; Weber and Springer, 1997), which influence the activity of the neutrophils (Kaplan et al., 2015). These aggregates modify the distribution of CD11b/CD18 (Mac-1 or CR3) on the neutrophils, by favoring their diapedesis in the inflamed vessels (Sreeramkumar et al., 2014) or in the inflamed pulmonary tissues (Valerio-Rojas et al., 2013). In these processes, the platelets exercise strong pro-inflammatory effects which damage the tissues. In fact, the platelet impoverishment in the animal models presenting pulmonary lesions improves tissue damage (Sreeramkumar et al., 2014). This process evokes the lowering of respiratory destress occurrences by the three anti-GPIIb/IIIa treatments used in this study. Effectively, despite the global lack of clinical efficacy of the two other drugs (ABX and EPT) compared with TIR, a reduction in pneumological signs was noted compared with the control animals having dive. At his step, we could suggest PLA could be formed in this DCS model, but it should be strictly demonstrated.

In parallel, EPT does not seem to limit hemoconcentration caused by the hyperbaric exposure. In other words, ABX and EPT have helped to maintain the hematocrit at its initial threshold. A capillary leak is described in severe cases of DCS in animals (Vallee et al., 2016; Cosnard et al., 2017) or humans (Gempp et al., 2013, 2014; Hibi et al., 2017), and it is likely that these two molecules have been able to contain it by inhibition of platelet aggregation (Hibi et al., 2017) or of the inflammatory syndrome which results from it (Luo et al., 2017).

### Why Would Tirofiban Be More Effective Than EPT and ABX?

Only TIR can contain platelet recruitment significantly as in principle; it has no action other than on GPIIb/IIIa, only a variation in platelet recruitment was expected. Furthermore, none of the molecules used has succeeded in limiting the decrease in the number of circulating leukocytes. Yet Thom et al. (2006) observed that TIR inhibits platelet-neutrophil aggregation. GPIIb/IIIa is also expressed on the leukocytes surface, and it participates in their activation (DeFranco et al., 2009).

**FIGURE 6:**
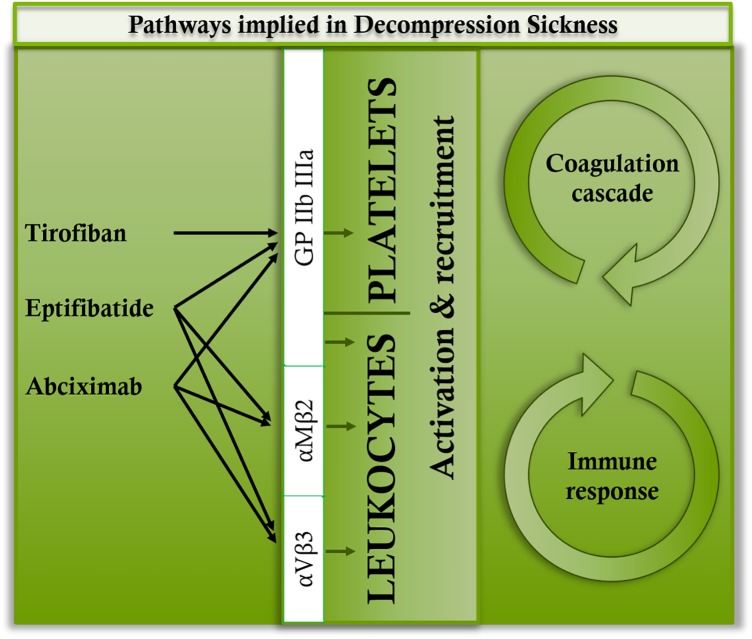
Main binding sites of tirofiban, abciximab, and eptifibatide.

As it is well known in DCS, the inflammation persists without the presence of bubble. Therefore, it seems likely that the leukocyte and platelet recruitment requires a parallel path (Pontier et al., 2011; Blatteau et al., 2015; de Maistre et al., 2016a,b; Vallee et al., 2016; Cosnard et al., 2017). Although platelet aggregation remains a significant phenomenon in DCS, as this experiment has demonstrated, the role of various anti-GPIIb/IIIa inhibitors and their receptors remains to be clarified. TIR is a low molecular weight molecule (> 1 KD) created to bind specifically and effectively to GPIIb/IIIa. It has shown itself to be highly effective in animals (Lynch et al., 1995). However, its bond is described as reversible and relatively weak (Bledzka et al., 2013). It does not interact with αVβ3 and αMβ2 (Coller, 1999b; Kereiakes et al., 2000; Lele et al., 2001). Its function in our experimentation should be essentially anti-thrombotic, considering the low variation in platelet counts (**Figure 5**). It has obviously been able to limit the capillary leakage but the mechanism, although it seems indirect, remains unclear. EPT is a very powerful inhibitor of the bond between fibrinogen and platelets, and has also been created specifically to bind to GPIIb/IIIa (Scarborough et al., 1993) even though there is contradictory data indicating that it could also bind to αVβ3 and αMβ2 (Lele et al., 2001; Goto et al., 2003) (**Figure 6**). For a peptide, EPT has a relatively long half-life in plasma (Coller, 1999b; Kereiakes et al., 2000; Lele et al., 2001). EPT remains the most widely used of the 3 αIIbβ3 antagonists approved by the FDA (Bledzka et al., 2013). At this concentration, it has not demonstrated anti-thrombotic or anti-inflammatory activity (in the way of stabilizing platelet or leukocyte counts) sufficiently effective against DCS although the respiratory distress signs diminished. ABX is a monoclonal antibody which can interact with integrins other than GPIIb/IIIa. ABX reacts with αVβ3 and αMβ2 (Mac-1, CD11b/CD18), a member of the integrin β2 sub-family of leukocyte integrins (Simon et al., 1997) (**Figure 6**). This cross-reactivity contrasts with other GPIIb/IIIa antagonists and may be responsible for the unique anti-inflammatory properties attributed to ABX (Coller, 1999a). This cross-reactivity distinguishes it from the other 2 GPIIb/IIIa antagonists, but there is no distinct proof that such cross-reactivity is either advantageous or prejudicial (Bledzka et al., 2013). Another unique characteristic of ABX is its prolonged association with platelets (Kereiakes et al., 2000). In this study, no efficient activity was shown by ABX, nor by EPT. The pulmonary signs have lessened but no anti-inflammatory activity (linked to cytokine levels) has been highlighted. It is likely that in this study, αVβ3 and/or αMβ2 could be blocked by TIR and EPT. However, the blocking of αVβ3 and αMβ2 should not hamper the anti-thrombotic activity of these substances. The inhibition of these inflammatory pathways in direct contact with the leukocytes would therefore be harmful insofar as it represents the body’s first line of defense against aggression (Jung and Yuki, 2016; Koutsogiannaki et al., 2017). Actually, αVβ3 and αMβ2 are expressed on the surface of neutrophils monocytes or natural killers (among others) and they represent, as GPIIb/IIIa, key points of their activation. For example, the expression of integrin αMβ2 (CD11b/Mac1, normally present on macrophages) and CXCR5, which binds chemokine CXCL13 (BLC-1), directs and maintains B1 cells in pleural or intestinal cavities as in B follicles of secondary lymphoid organs (DeFranco et al., 2009). So, we wonder whether inhibiting these communications is not a wrong approach for limiting mortality in DCS. TIR is therefore the only drug in this study where BLC-1 (CXCL13 in human) levels increased. BLC-1 is chemo attractive, and it is supposed to regulate, among others, the follicular activity of B1 cells in the secondary lymphatic organs, in order to help them to be co-stimulated by T helpers, and finally initiate the humoral immunity answer. But inhibiting αVβ3 modulates the T helpers cells response (Wang et al., 2014) and it could so inhibit the B cells activation. B1 cells also react to Toll-like receptors (TLRs) ligands by secretion of antibodies (DeFranco et al., 2009).

Also, the particular role of platelets in inflammation should be stressed. In 2004, the Japanese team of Yokoyama showed the presence of TLR 1 and 6 on the surface of platelets, with possible involvement of the latter in the process of atherosclerosis (Shiraki et al., 2004). In the same year, the work of another team completed these observations by demonstrating TLR 2, 4 and 9 both on their platelet surfaces and also in the cytoplasm; and by demonstrating that their expression could be modulated depending on the activation status of the platelets (Cognasse et al., 2005). The characteristic expression of TLRs on the surface of platelets is an argument with weight for considering these cells as participants in immunity. As for FcγRIIa (Cassel et al., 1993), the high number of circulating platelets carrying immunoreceptors suggests to consider them as sentinels able to detect danger. The platelet TLR9 has recently been reported for binding carboxyalkyl-pyrrole, a product resulting from the combination of the oxidation products of polyunsaturated fatty acids and proteins, considered to be a danger signal in oxidative stress (Panigrahi et al., 2013). In the platelet, this bond triggers aggregations and degranulation. Consequently, in addition to being sentinels for immune response, the platelets would be sensors for internal danger signals, particularly by TLR9. Furthermore, we have just shown an increase in the amount of circulating mitochondrial DNA in DCS (Cosnard et al., 2017), and we know that this binds promptly to TLR-9s to induce inflammation.

### Weighting and Limitations

Compared to the first study testing ABX at the same concentration in DCS (Lambrechts et al., 2015), the number of animals in our study is twice higher, but we used isoflurane to limit the stress of the injection, as recommended by the ethical committee. The result of the first study mainly focused on basic symptoms of DCS. No blood cell counts, including platelet count, were conducted. They found no significant difference in PF4 (as in this study) or VWF levels compared to the control dive group, which can be attributed to the ABX effect, but Tbars were decreased.

In our study, isoflurane was used in all groups (for 2 min maximum) of this study and its effects must be the same in all of them. DCS occurrence (in term of a percent) seems to the same in the Ctrl D group than those of our previous studies (without using isoflurane) using the same hyperbaric protocol. Nonetheless, we could consider inhibition by isoflurane on aIIbβ3 (or GPIIb/IIIa) could disappear rapidly (especially at the decompression time: more than 1 h after the inhalation) or (if not) add to those effects desired for anti-GPIIb/IIIa drugs. If right, these results provide a potential mechanism for the immunomodulatory properties of isoflurane and an interaction with anti-GPIIb/IIIa drugs, which would confirm, on the one hand, the involvement of platelet/leukocyte communication in the DCS (and especially in its prevention), as it exists elsewhere (Yuki et al., 2008, 2013; Carbo et al., 2013), and on the other hand that the coagulant role of platelets in DCS is perhaps secondary. This remains to be highlighted.

We could also regret the lack of study at the tissue level, to confirm diapedesis or cell destruction more precisely for example. The mean platelet volume could have help in the interpretation as it did in a previous article (Vallee et al., 2016). Nonetheless, this study permitted to test drugs that should not have been allowed to test directly in humans, but which opened a hypothesis that should be confirmed in humans.

## Conclusion

This protocol generates DCS symptoms with pro-thrombotic and pro-inflammatory phenomena, accompanied by neurological and respiratory distress. Although the three anti-GPIIb/IIIa agents used tended to limit respiratory distress in the rats, only TIR significantly reduced DCS occurrence. TIR is specific to GPIIb/IIIa whereas EPT and ABX could inhibit αVβ3 and αMβ2 involved in communication with the immune system. While inhibiting GPIIb/IIIa could highlight a platelet-dependent inflammatory pathway that improves DCS outcomes, we wonder whether inhibiting the αVβ3 and αMβ2 communications is not a wrong approach for limiting mortality in DCS. The immune response in DCS seems essential, but it remains to be explored.

## Author Contributions

KL and NV performed the conception and design of research. KL, SdM, and NV performed the experiments. KL and NV analyzed the data. KL, SdM, JA, J-EB, J-JR, and NV interpreted the results of experiments. NV prepared the figures. KL and NV drafted, edited, and revised the manuscript. KL, SdM, JA, J-EB, J-JR, and NV approved the final version of manuscript.

## Conflict of Interest Statement

The authors have no competing interest to disclose in this study. The results of the present study do not constitute endorsement by ACSM. The results of the study are presented clearly, honestly, and without fabrication, falsification, or inappropriate data manipulation.

## Abbreviations

ABX: abciximab
Ctrl: control
DCS: decompression sickness
EPT: eptifibatide
GPIIbIIIa: glycoprotein IIb/IIIa
ND: non-divers
TIR: tirofiban
TLR: toll-like receptor
